# Human Brown Adipose Tissue Estimated With Magnetic Resonance Imaging Undergoes Changes in Composition After Cold Exposure: An *in vivo* MRI Study in Healthy Volunteers

**DOI:** 10.3389/fendo.2019.00898

**Published:** 2020-01-09

**Authors:** Gustavo Abreu-Vieira, Aashley S. D. Sardjoe Mishre, Jedrzej Burakiewicz, Laura G. M. Janssen, Kimberly J. Nahon, Jari A. van der Eijk, Titia T. Riem, Mariëtte R. Boon, Oleh Dzyubachyk, Andrew G. Webb, Patrick C. N. Rensen, Hermien E. Kan

**Affiliations:** ^1^Division of Endocrinology and Einthoven Laboratory for Experimental Vascular Medicine, Department of Medicine, Leiden University Medical Center, Leiden, Netherlands; ^2^Department of Radiology, C.J. Gorter Center for High Field MRI, Leiden University Medical Center, Leiden, Netherlands; ^3^Division of Image Processing (LKEB), Department of Radiology, Leiden University Medical Center, Leiden, Netherlands

**Keywords:** brown adipose tissue, lipid metabolism, cold exposure, thermogenesis, magnetic resonance imaging, fat fraction

## Abstract

**Aim:** Magnetic resonance imaging (MRI) is increasingly being used to evaluate brown adipose tissue (BAT) function. Reports on the extent and direction of cold-induced changes in MRI fat fraction and estimated BAT volume vary between studies. Here, we aimed to explore the effect of different fat fraction threshold ranges on outcomes measured by MRI. Moreover, we aimed to investigate the effect of cold exposure on estimated BAT mass and energy content.

**Methods:** The effects of cold exposure at different fat fraction thresholding levels were analyzed in the supraclavicular adipose depot of nine adult males. MRI data were reconstructed, co-registered and analyzed in two ways. First, we analyzed cold-induced changes in fat fraction, T2* relaxation time, volume, mass, and energy of the entire supraclavicular adipose depot at different fat fraction threshold levels. As a control, we assessed fat fraction differences of deltoid subcutaneous adipose tissue (SAT). Second, a local analysis was performed to study changes in fat fraction and T2* on a voxel-level. Thermoneutral and post-cooling data were compared using paired-sample *t*-tests (*p* < 0.05).

**Results:** Global analysis unveiled that the largest cold-induced change in fat fraction occurred within a thermoneutral fat fraction range of 30–100% (−3.5 ± 1.9%), without changing the estimated BAT volume. However, the largest cold-induced changes in estimated BAT volume were observed when applying a thermoneutral fat fraction range of 70–100% (−3.8 ± 2.6%). No changes were observed for the deltoid SAT fat fractions. Tissue energy content was reduced from 126 ± 33 to 121 ± 30 kcal, when using a 30–100% fat fraction range, and also depended on different fat fraction thresholds. Voxel-wise analysis showed that while cold exposure changed the fat fraction across nearly all thermoneutral fat fractions, decreases were most pronounced at high thermoneutral fat fractions.

**Conclusion:** Cold-induced changes in fat fraction occurred over the entire range of thermoneutral fat fractions, and were especially found in lipid-rich regions of the supraclavicular adipose depot. Due to the variability in response between lipid-rich and lipid-poor regions, care should be taken when applying fat fraction thresholds for MRI BAT analysis.

## Introduction

The main function of brown adipose tissue (BAT) is to convert chemical energy stored within lipids into thermal energy (heat). Exposure to low temperatures is the main physiological stimulus for BAT activation (1). Upon adrenergic stimulation by sympathetic nerves, intracellular lipolysis takes place within brown adipocytes (2), and the resulting free fatty acids bind to uncoupling protein 1 (UCP1), which, in turn, functions as a molecular gate that dissipates the generated mitochondrial proton gradient as heat. To replenish the intracellular lipid stores, BAT takes up glucose and fatty acids from the systemic circulation (3, 4). In rodents, visualization of BAT by magnetic resonance imaging (MRI) was first reported almost three decades ago (5), and soon the technique was shown to accurately reflect the tissue structure (6) as well as histological changes due to temperature acclimatization (7). More recently, with research being expanded toward human physiology, several studies explored this ionizing-radiation-free method with the aim of understanding BAT function (8). From preclinical models it is known that the chemically-assessed fat content of tissues matches the fat mass estimated by MRI (9) and that fat fraction (FF) correlates negatively with the amount of UCP1-expressing cells in BAT (10) and positively with adipocyte size (11). In the intrascapular BAT of rodents kept on regular chow and at room temperature (circa 21°C), MRI estimations of FF vary between 20 and 50%, depending on the depth of the tissue (12). However, FF can reach up to almost 80% when animals are kept at thermoneutrality (13). In infants, BAT resembles the classic intrascapular depot found in rodents, both in morphology and function (14). In adults, however, there is a remarkable lack of easily distinguishable borders for e.g., the supraclavicular depot, which makes it difficult for a consensus to be reached on the optimal FF thresholds that should be used for specific BAT imaging (15). As a consequence, FF within human BAT has variously been described as circa 60% (16), 65% (17), 80% (18, 19), and 94% (20) in elderly adults and different FF threshold levels have been used to segment BAT (19, 21–24). Only one recent study explored the effect of specific FF threshold levels (0–100, 40–100, and 50–100%) on the cold-induced response in FF (25), but no analyses on other MR outcome parameters were explored. The relaxation time T2* has also been studied as an indirect MRI measure of BAT activity (16, 21, 24, 26, 27). It has been demonstrated that the T2* of BAT is shorter compared to white adipose tissue (WAT), which is most likely due to the abundant iron-rich mitochondria present in brown adipocytes. Cold-induced BAT activation increases oxygen consumption due to increased metabolic activity, which in turn increases blood perfusion. The latter increases T2*, whereas oxygen consumption shortens T2* (21). Different reports exist on the direction of changes in T2* during cold exposure, most likely due to these conflicting effects (17, 28, 29). BAT is naturally heterogeneous: on a molecular scale, this is manifested in differences in UCP-1 protein expression of adjacent cells, which after immunostaining create a multicolored pattern termed the “harlequin phenomenon” (30). The lack of homogeneity between adipose tissue depots within a single organism has also been noted at the functional level (31–33). Although structural heterogeneity has been noted in BAT imaging studies (34–37), it is generally seen as a confounding factor. Moreover, while the major goal of BAT medical research is to understand and manipulate energy fluxes, the quantification of tissue mass as caloric equivalents is rare. There are a few interesting examples of such a concept being applied, e.g., by matching body composition to potential energy storages and predicting whole-body energy expenditure (38, 39) or inferences concerning BAT energy uptake by estimating the energy content in labeled macromolecules (40). To our knowledge, however, an estimation of BAT energy storages *in vivo* has not been performed yet. Given the importance of BAT in current metabolic research, we aimed to explore the effect of different fat fraction threshold ranges on multiple outcomes measured by MRI. Moreover, for the first time, we aimed to investigate the effect of cold exposure on BAT mass and energy content. To this end, we first assessed estimated BAT volumes at thermoneutral and cold conditions to establish a lower FF threshold for the exclusion of non-fatty voxels. Subsequently, we determined estimated BAT volume, FF, T2*, mass and energy content, and explored the effect of different FF thresholds on these parameters. Finally, we assessed local changes in FF and T2* upon cold exposure on a voxel-level. We demonstrate the importance of the high-lipid areas of the tissue and suggest that the conceptual framework of this work could further aid investigations on BAT as a target for obesity and metabolic disorders in humans.

## Materials and Methods

### Subjects

Ten healthy, non-smoking, lean (BMI 18–25 kg/m^2^) Europid male volunteers, born in the Netherlands and aged between 18 and 30 years, were recruited as part of a larger intervention study that investigated the effect of cold exposure and the β3-receptor agonist mirabegron on BAT (Clinical Trials number: NCT03012113). The study was conducted in accordance with the principles of the revised Declaration of Helsinki (41) and with approval from the local medical ethics committee. Exclusion criteria were recent excessive weight change (>3 kg within the last 3 months), vigorous exercise, use of any medication known to affect lipid and/or glucose metabolism, BAT activity, cardiac function or QT interval time, smoking and any relevant chronic disease. Contraindications for undergoing an MRI scan were the presence of non-MR-safe metal implants or objects in the body (i.e., a pacemaker, neurostimulator, hydrocephalus or drug pump, non-removable hearing aid or large recent tattoos), and a history of claustrophobia, tinnitus, or hyperacusis.

### Study Design and BAT Activation Protocol

Subjects were instructed to withhold from alcohol and caffeine for 24 h and to fast overnight for 10 h, prior to the experiment. Subjects remained fasted until the end of the experiment. To activate BAT, a personalized cooling protocol was conducted as previously described (42). Each subject was placed between water-perfused temperature-controlled mattresses with water initially circulating at 32°C. The water temperature was gradually reduced during the first hour until reaching 9°C or reporting of shivering by the subject. In either case the temperature was raised by 3°C and the subject laid for one additional hour under these conditions. In the case of renewed shivering, the temperature was raised slightly to stop shivering and to assure that BAT remained the dominant source of heat production (2). MRI scans were acquired before and after the cooling protocol on a 3 T MRI scanner (Philips Ingenia, Philips Healthcare, Best, The Netherlands). Subjects were positioned supine and head-first in the scanner. Scans were conducted at the same time of day in all participants (before cooling: in the morning, after cooling: in the afternoon).

### Image Acquisition

**Figure 1:**
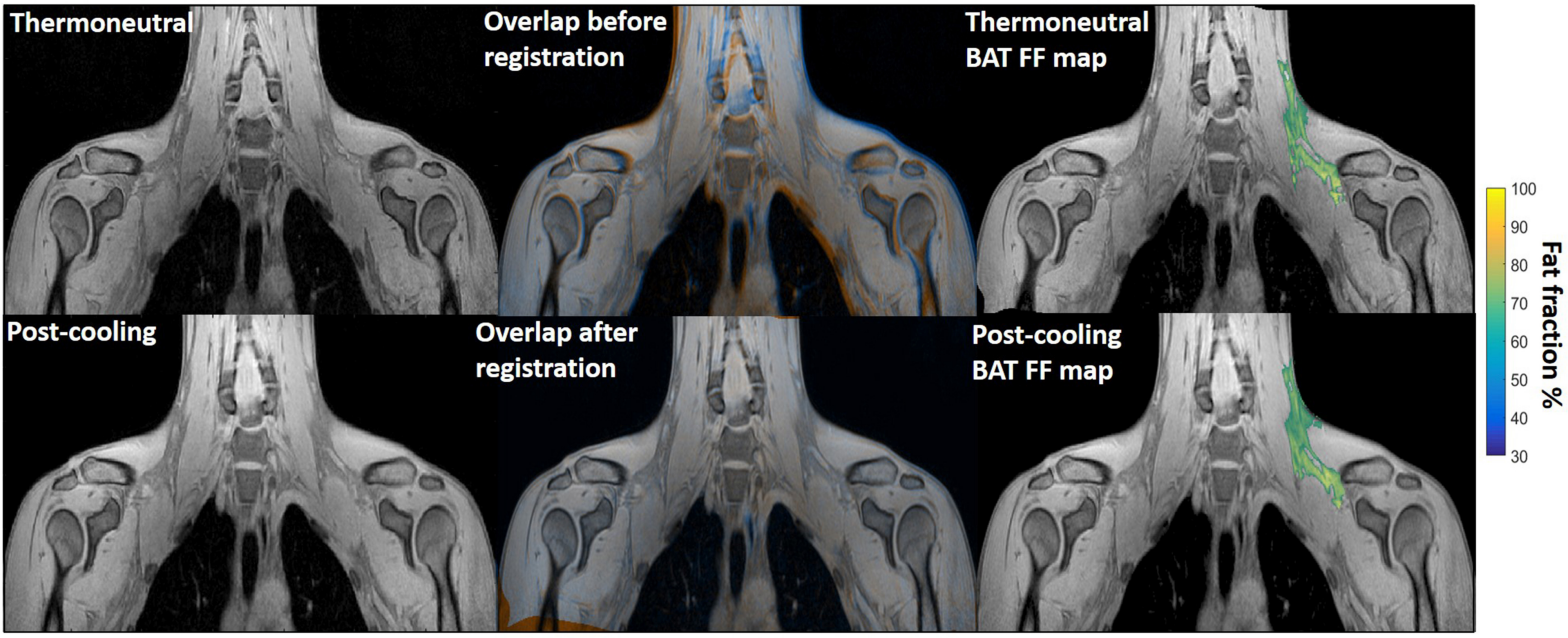
Example of image registration and a reconstructed fat fraction map before and after cooling. The first column shows thermoneutral and post-cooling images (one slice from the first echo in the acquisition). In the second column, the overlay of the same images before (top) and after registration (bottom) is shown. The images are colored orange (thermoneutral) and blue (post-cooling) for better visualization of differences between the scans. The third column shows the thermoneutral and post-cooling fat fraction maps of the supraclavicular adipose depot, overlaid on the corresponding images. Lipid content in the supraclavicular region is color-mapped over a 30–100% fat fraction range.

A three-dimensional six-point chemical-shift encoded gradient-echo acquisition using a 16-channel anterior array, 12-channel posterior array and the posterior section of the 16-channel head and neck coil was used to image the supraclavicular adipose depot (Figure 1). The following imaging parameters were used: repetition time TR = 15 ms, first echo time TE = 1.98 ms, echo time separation ΔTE = 1.75 ms, flip angle = 8°, field-of-view of 480 × 300 × 90 mm^3^ (Right-Left, Foot-Head, Anterior-Posterior), 1.1 mm isotropic resolution, four signal averages. Averaging was done post-acquisition; in the case of significant subject motion the corresponding averages were rejected. Bulk motion due to either shivering or subject discomfort was the major source of motion. The total imaging time was 12 min. To increase the reproducibility of subject positioning, the participants were asked to reach as far as possible with their fingers toward their feet after being placed on the scanner table and to relax their shoulders afterwards.

### Data Reconstruction and Analysis

#### Data Reconstruction

Quantitative water and fat images were reconstructed off-line using an in-house water-fat separation algorithm based on the known frequencies of the multi-peak fat spectrum and assuming a mono-exponential T2*, combined with a region-growing scheme to mitigate strong main field inhomogeneity effects. Initially, a low-resolution reconstruction was performed by using an estimate for the main magnetic field inhomogeneity. Subsequently, a region growing scheme was used to extrapolate the solution from correctly reconstructed parts in order to acquire the reconstructed water and fat images at high resolution (43–46). FF maps were generated according to the following equation, where x, y, and z denote the position of a voxel in the image.

$$\begin{array}{l} Signal\;fat\;fraction\left(x,y,z\right)\\ =\frac{Signal_{Fat}\left(x,y,z\right)}{Signal_{Fat}\left(x,y,z\right)\;+\;Signal_{Water}\left(x,y,z\right)} \end{array}$$

#### Image Registration and ROI Delineation

Registration was performed using the open-source image registration toolbox Elastix (47, 48). The first echo of the thermoneutral image stack was registered to that of the post-cooling stack by first pre-aligning them in an affine manner followed by deformable registration with a three-dimensional B-spline transform with a 10 × 10 × 10 mm^3^ grid. In both cases, an adaptive stochastic gradient descent with two resolutions for optimization and Mattes mutual information was used as the similarity measure. Region masks, defined as the sampled part of each image stack, were used during the registration. The parameter files that were used for performing the registration can be downloaded from http://elastix.bigr.nl/wiki/index.php/Par0048. Regions of interest (ROIs) encompassing the known location of the left supraclavicular adipose depot (49) (Figure 1) were drawn manually on the thermoneutral scans by one observer. Additionally, to ensure that potential changes in FF of the supraclavicular BAT depot were specific to this region, regions of interest comprising deltoid subcutaneous adipose tissue (SAT) were manually delineated on both the thermoneutral and post-cooling scans (Supplemental Figure S1). To exclude potential bias caused by the direction of registration, we also performed the registration in the reverse direction (post-cooling → thermoneutral) and obtained results (not shown) that were virtually identical to the ones reported below.

#### Data Analysis

Cold-induced changes to the supraclavicular adipose depot were assessed using two complementary analyses. First, changes in FF, T2*, volume, mass, and energy content of the supraclavicular adipose depot were assessed using a global analysis. As this analysis only uses the deformation field for ROI mapping on the post-cooling image, this allows not only assessment of FF, but also any changes in estimated BAT volume. Assessment of BAT volume was recently shown to be highly dependent on segmentation criteria in [^18^F]FDG PET-CT studies (50). Therefore, we decided to explore the influence of FF segmentation criteria on both estimated BAT volume and FF using MRI.

The estimated BAT volume was determined by multiplying the volume of a single voxel (0.548 μL) by the number of voxels that fall within a certain fat fraction segmentation range (e.g., 30–100% FF). For example: when using a 30–100% FF segmentation threshold range, 93275 voxels were segmented from the thermoneutral image. Multiplied by the volume of a single voxel (0.548 μL), the estimated BAT volume would be 51 mL. Data from this analysis were also used to explore different FF thresholds. Secondly, we performed a local analysis to study changes in FF and the T2* relaxation time on a voxel-level. As this method directly deforms the thermoneutral images and ROIs to post-cooling image coordinates, no conclusions regarding the true volume can be inferred Details of the methods are outlined below. Due to excessive movement during image acquisition, MRI data from one participant could not be reconstructed and were excluded from all analyses.

- **Global analysis : FF**_**Glob**_**, FF**_**SAT**_**,** $\text{T}2_{\text{Glob}}^{*}$, and Vol_**BAT**_
  Global analysis of supraclavicular adipose tissue FF (FF_Glob_), T2* relaxation time (${\text{T}2}_{\text{Glob}}^{*}$) and estimated BAT volume (Vol_BAT_) was performed by mapping the defined ROIs to the post-cooling image coordinates. To this end, the calculated deformation field from the registration was used to transform the thermoneutral ROIs to the post-cooling scan coordinates. The deformation field of the ROIs was converted to the floating point image type. This enabled performing the analysis on raw (non-interpolated) data. The distribution of thermoneutral and post-cooling Vol_BAT_ across the FF range was assessed using volume histograms with FF bins of 0.5%. This was then assessed statistically by determining at which FF ranges (10% intervals), estimated BAT volume was significantly changed after cold exposure. To explore the effect of different upper and lower FF thresholds for BAT analysis, cold-induced changes in Vol_BAT_, FF_Glob_, and ${\text{T}2}_{\text{Glob}}^{*}$ were quantified at all FF threshold options. To illustrate these effects, we tested for specific FF ranges: 30–100, 50–100, and 70–100% whether Vol_BAT_, FF_Glob_, and ${\text{T}2}_{\text{Glob}}^{*}$ changed significantly after cold exposure. Voxels below the selected lower FF thresholds (i.e., 30, 50, or 70%) were excluded in both the thermoneutral and post-cooling ROIs. By plotting the ROIs using different lower FF segmentation thresholds, we observed that voxels within a 10–30% FF interval were mostly located at the boundaries of the supraclavicular adipose depot, which are adjacent to muscle (Supplemental Figure S2). Therefore, to avoid inclusion of non-fatty tissue and minimize partial volume effects, a lower FF threshold of 30% was adopted for the subsequent analyses. ROIs comprising deltoid SAT were manually delineated on both thermoneutral and post-cooling scans to preclude analysis bias arising from difficulty registering ROIs located at the interface of tissue and air (Supplemental Figure S1). The average FF of the deltoid SAT depots (FF_SAT_) was determined using a 70–100% FF interval before and after cooling to avoid voxels containing muscle and air and to minimize partial volume effects.
- **Global analysis: estimation of BAT mass and energy content**
  To estimate BAT mass and energy content, the FF was used to calculate water and fat mass, and, subsequently, the total tissue energy was estimated similarly to (38–40). 1 μl of lipid was assumed to represent 0.92 mg in mass, corresponding to 9.4 × 10^−3^ kcal. Lean mass measurements were derived from the water MR signal and represent a combination of water-bound structures, such as proteins, glucose and intra- extracellular fluids. Lean mass of 1 μl corresponded to 1.06 mg and 1.0 × 10^−3^ kcal, correspondingly. Energy variation and lean/fat mass changes were calculated from the FF. Therefore, a voxel of 1 μl with a FF of 50% is equivalent to 0.5 μl lean mass and 0.5 μl fat, which, after adjustments for density, represented 0.455 mg fat and 0.540 mg lean mass.
- **Voxel-wise analysis: FF**_**Loc**_ **and** $\text{T}2_{\text{Loc}}^{*}$
  For voxel-wise analysis of the supraclavicular adipose depot, the deformation field from the registration was used to transform the thermoneutral ROIs, FF and T2* maps to the post-cooling image coordinates to compare the FF and T2* on a voxel-level (FF_Loc_ and ${\text{T}2}_{\text{Loc}}^{*}$). To compensate for potential bias due to interpolation of the moving image and small-scale inconsistencies between the co-registered images, each voxel of both thermoneutral and post-cooling image stacks was assigned a mean value from its 3 × 3 voxel neighborhood.

FF maps were generated to visualize FF composition changes across the supraclavicular adipose depot on a voxel-wise level. Cold-induced FF changes on a voxel level (FF _Loc_) were further studied using two-dimensional joint histograms. In these plots, for every voxel its initial FF was plotted against its change in FF after cold exposure, and the number of voxels belonging to each combination was added to represent the counts (color scale). Similar voxel density plots were used to assess (i) the relation between thermoneutral ${\text{T}2}_{\text{Loc}}^{*}$ and FF_Loc_, (ii) the relation between Δ${\text{T}2}_{\text{Loc}}^{*}$ after cold exposure and thermoneutral FF measurements, and (iii) the relation between Δ${\text{T}2}_{\text{Loc}}^{*}$ and ΔFF_Loc_ after cold exposure. The distributions of thermoneutral FF_Loc_, ΔFF_Loc_, and Δ${\text{T}2}_{\text{Loc}}^{*}$ after cold exposure were assessed using K-means clustering. The Elbow method (51) was used to obtain the optimal cluster number by evaluating the percentage of explained variance as a function of the number of clusters. The explained variance percentage was determined as the ratio of the between-group variance to the total variance. In general, when the explained variance is plotted against cluster number, the first few clusters will add information (explain variance), so these can be observed as jumps from one k-value to another. However, at a certain k-value little information is added, which results in a knee point. For analyzing the voxel distributions, the optimal k-value was determined by visual inspection and implementing a 95% explained variance cut-off value.

#### Statistical Analysis

Data were tested for a normal distribution according to the Shapiro-Wilk test. For the global analysis, comparisons between thermoneutral and post-cooling data were performed by paired Student's *t*-tests with results deemed statistically significant when *p* < 0.05. No correction for multiple comparisons was performed. For the local analysis we used a voxel-wise comparison, and performed k-means clustering for the analysis. As this approach uses an unsupervised learning algorithm that simply visualizes underlying clusters in the voxel distribution without providing any details regarding the significance of the different clusters, no correction for multiple comparisons is needed (51). Linear regression was used to assess the relation between supraclavicular adipose tissue mass and volume using a 0.05 significance level and the R-squared is given. Data analysis including statistical analysis was performed in MATLAB (version R2018b). Data are presented as mean ± SEM.

## Results

### Volumetric Changes in Estimated BAT Volume After Cold-Exposure

**Figure 2:**
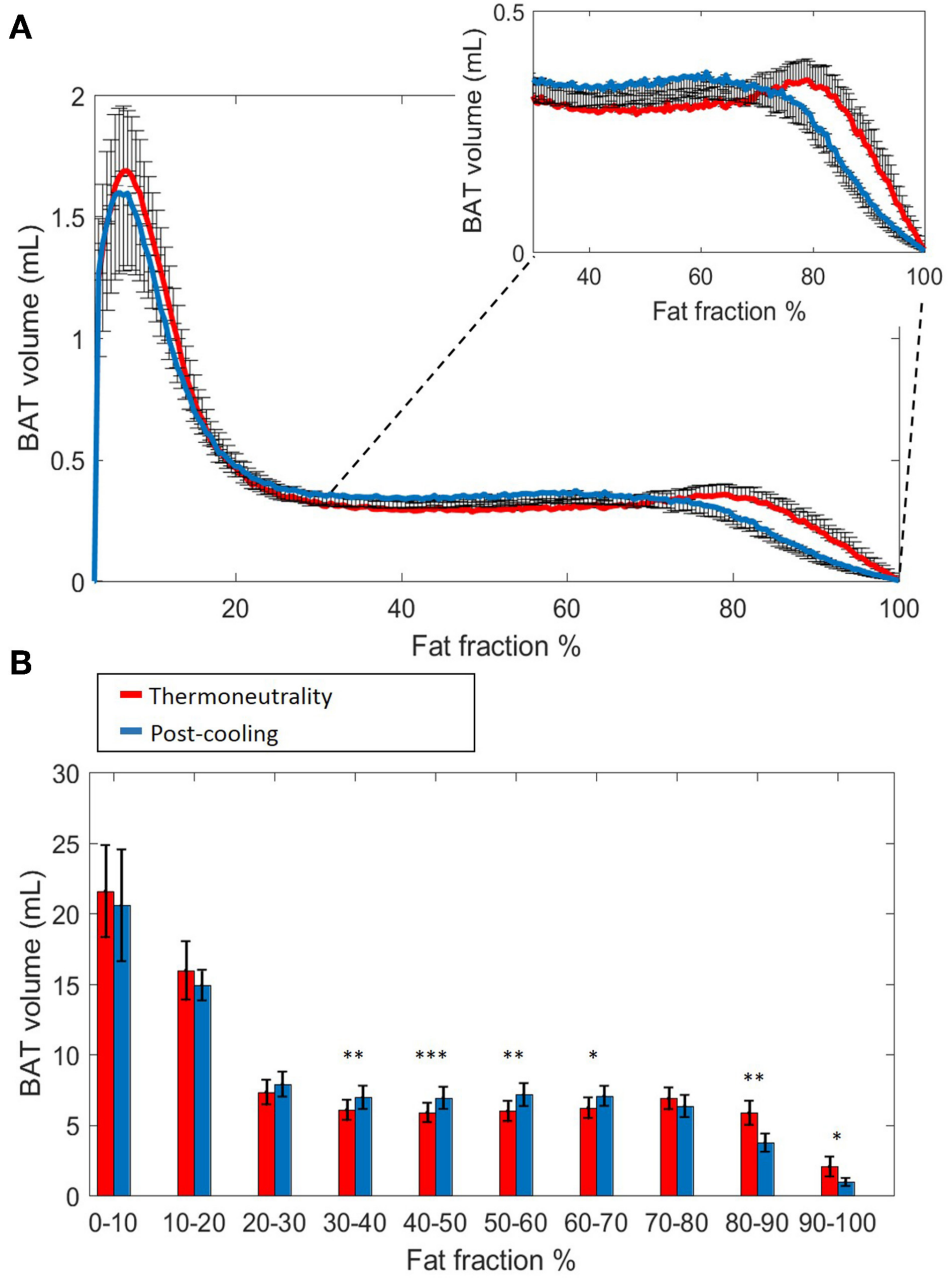
Estimated volumetric BAT analysis. Thermoneutral and post-cooling volume histograms as a function of fat fraction with bin size 0.5%: thermoneutral volumes are shown in red and post-cooling volumes in blue **(A)**. Cold-induced volume changes plotted as a function of fat fractions (10% FF interval) **(B)**. Data are represented as mean ± SEM for *n* = 9. In **(B)**, a paired sample *t*-test was used to analyze the changes in volume after cold exposure. *p < 0.05, **p < 0.01, and ***p < 0.001.

**Figure 3:**
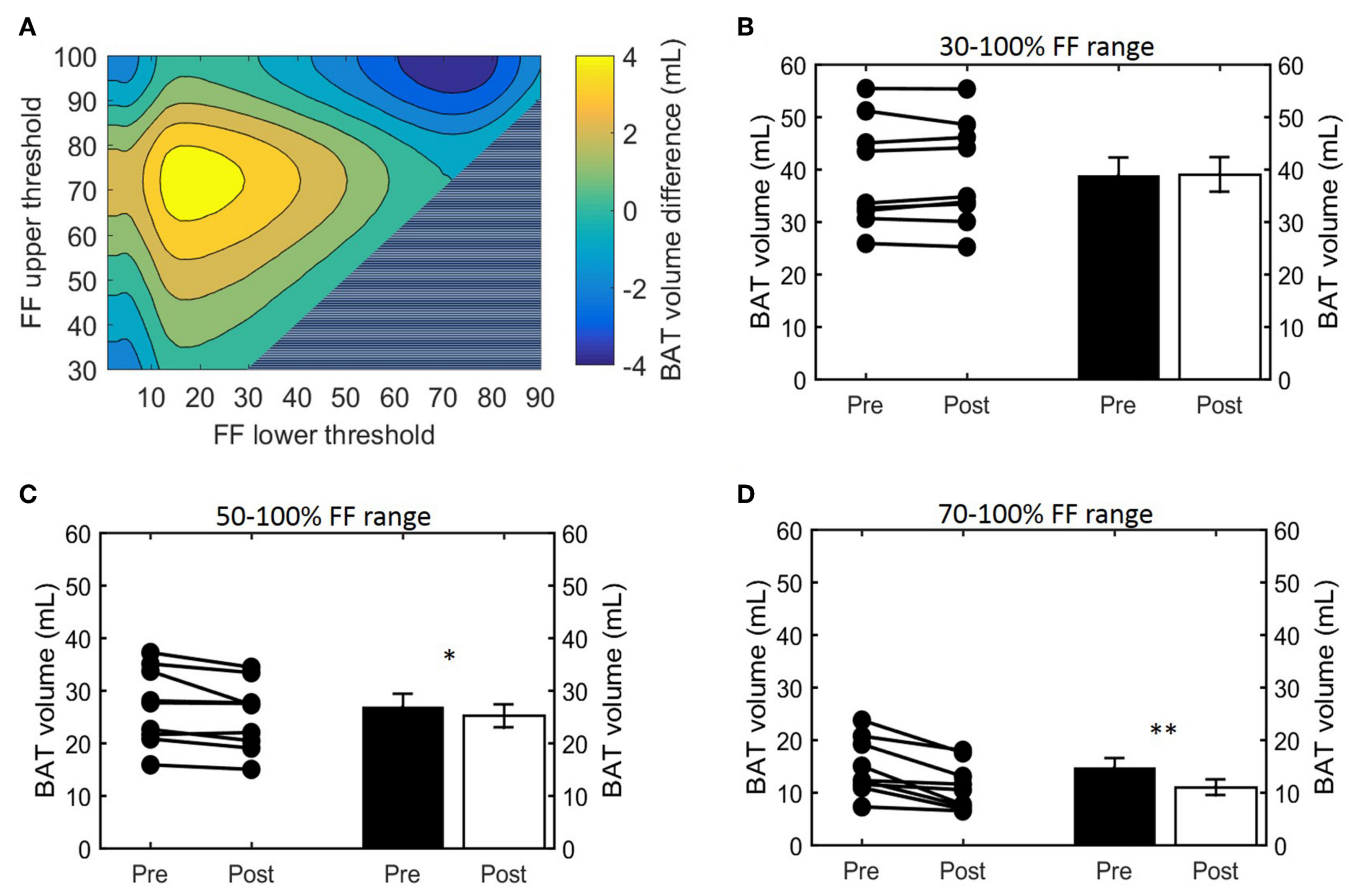
Effect of FF thresholds on estimated BAT volume differences. Heatmap of the effect of different FF segmentation thresholds on estimated BAT volume differences after cooling. The color (second y-axis) depicts the estimated BAT volume difference for each lower (x-axis) and upper left (y-axis) threshold. The largest decrease in estimated BAT volume is present with a lower threshold of 72% and no upper threshold. The triangle in the lower right corner indicates invalid FF threshold options, as we implemented a minimum FF threshold of 30%. **(A)** Cold-induced volume changes analyzed using the paired sample *t*-test (*p < 0.05, **p < 0.01) at different threshold ranges: 30–100% **(B)**, 50–100% **(C)**, and 70–100% **(D)**. Data is represented as mean ± SEM for all participants (*n* = 9).

Histogram analysis of the changes in Vol_BAT_ showed an overall shift of the estimated post-cooling BAT volume from higher FFs toward lower FFs (Figure 2A). When binned into 10% FF intervals, this resulted in significant increases in estimated BAT volume above a FF of 30%, while the estimated BAT volume was significantly decreased above a FF of 80% (Figure 2B). Interestingly, Vol_BAT_ did not change significantly within the 70–80% FF range, which is at the intersection of the thermoneutral and post-cooling histograms (inset of Figure 2A). The effect of different FF threshold options on cold-induced changes in Vol_BAT_ is shown in Figure 3A. For a lower FF threshold of 30% and upper FF threshold of 100%, no clear change in Vol_BAT_ occurred. However, with increasing lower FF threshold values, Vol_BAT_ decreased upon cold exposure. This was subsequently tested for statistical significance for FF ranges with a relatively low (30–100%), intermediate (50–100%), and high (70–100%) lower threshold. For the broadest FF range (30–100%), no significant change was detected in Vol_BAT_ after cold exposure (Figure 3B). For the intermediate FF range (50–100%), Vol_BAT_ lowered from 26.9 ± 2.4 to 25.2 ± 2.2 mL (−1.8%; *p* = 0.031, Figure 3C) after cold exposure. For the 70–100% FF range, Vol_BAT_ decreased from 14.7 ± 1.8 to 11.0 ± 1.5 mL (−3.8%; *p* = 0.0022, Figure 3D) after cold exposure.

### FF_SAT_ and the Effect of FF Thresholds on Global FF and T2*

**Figure 4:**
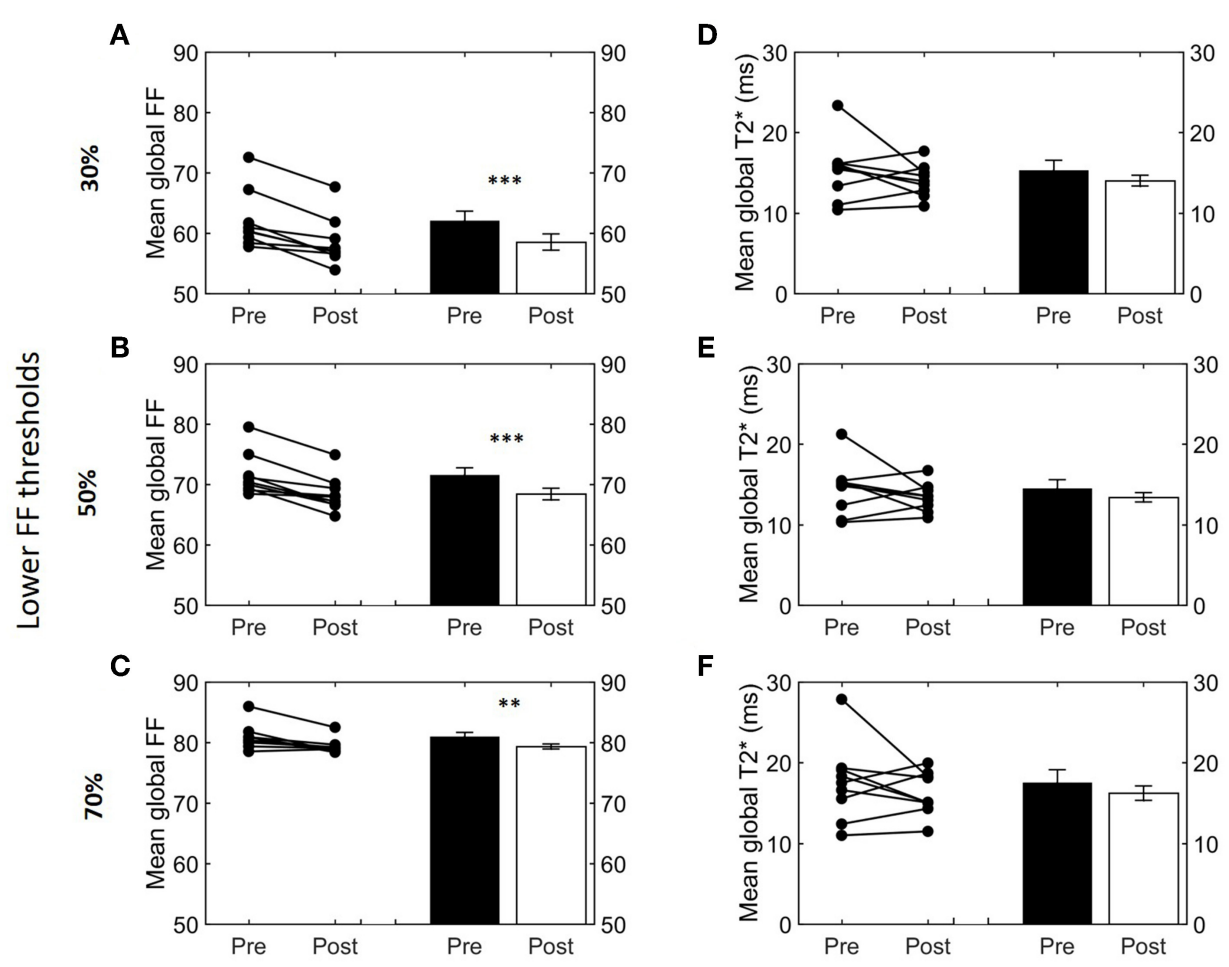
Effect of different FF thresholds on global supraclavicular adipose tissue FF and T2*. Cold-induced FF and T2* changes analyzed using the paired *t*-test at different threshold ranges: 30–100% **(A,D)**, 50–100% **(B,E)**, and 70–100% **(C,F)**. Data are represented as mean ± SEM for *n* = 9. The paired sample *t*-test was used to analyze the changes in volume after cold exposure (**p < 0.01 and ***p < 0.001).

Next, we studied how lower and upper FF thresholds affected the cold-induced change in FF_Glob_ (ΔFF_Glob_; post-cooling minus pre-cooling) and FF_SAT_ (ΔFF_SAT_; post-cooling minus pre-cooling), as well as ${\text{T}2}_{\text{Glob}}^{*}$ (Δ${\text{T}2}_{\text{Glob}}^{*}$; post-cooling minus pre-cooling). The largest decrease in FF_Glob_ occurred at a lower FF threshold of 34% and upper FF threshold of 100% (Supplemental Figure S3). This decrease in FF became smaller when shifting the lower FF threshold toward higher values. This was further tested for statistical significance for the following FF ranges: 30–100, 50–100, and 70–100%. When applying the 30–100% FF range, FF_Glob_ decreased from 62.0 ± 1.6 to 58.5 ± 1.3% (−3.5%; *p* = 5.0e-4, Figure 4A). With an intermediate threshold of 50–100%, FF_Glob_ decreased from 71.6 ± 1.2 to 68.4 ± 1.0% (−3.2%; 5.6e-4, Figure 4B). When a lower threshold of 70% was assumed, FF_Glob_ decreased from 81.0 ± 0.7 to 79.3 ± 0.4% (−1.6%; *p* = 0.006, Figure 4C). In contrast, no significant changes were noted in FF_SAT_ after cold exposure (Supplemental Figure S1B). For ${\text{T}2}_{\text{Glob}}^{*}$, no clear changes were seen as a function of different threshold options (Supplemental Figure S3, Figures 4D–F).

### Estimation of BAT Lipid and Lean Mass After Cold

**Figure 5:**
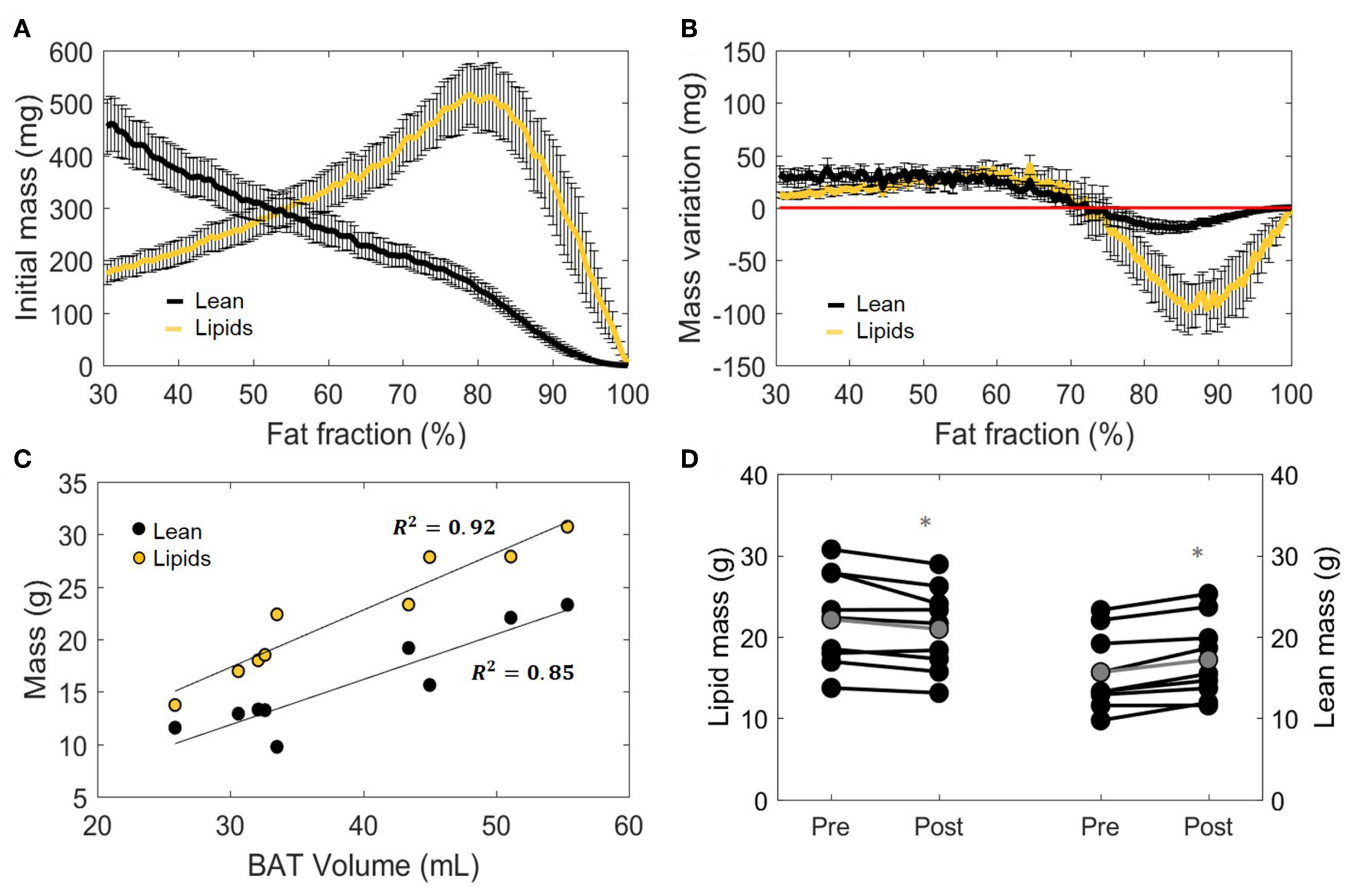
Distinction between lean and lipid masses within supraclavicular adipose tissue. Lean and lipid masses were estimated as described in the “Methods” section and represented as a function of their specific fat fractions **(A)**. Cold exposure decreased both lean and fat masses to in the upper fat fractions (above 70%) and slightly increased these in the lower fat fractions **(B)**. **(C)** Correlation between total estimated BAT volume and lipid or lean mass analyzed using linear regression (*R*^2^ is reported). Change in total lipid and lean mass after cold exposure, analyzed with the paired sample *t*-test **(D)**. Data in **(A,B,D)** represent mean ± SEM for *n* = 9 volunteers. *p < 0.05.

Having defined the effect of cold exposure on Vol_BAT_, FF_Glob_, and ${\text{T}2}_{\text{Glob}}^{*}$, we set out to characterize the subtle changes that take place within the tissue composition. Supraclavicular adipose tissue is composed of two compartments distinguishable by MRI: fat mass and lean mass. While fat mass comprises the accumulated lipid droplets, lean mass corresponds to water-rich structures, a broad category that includes blood, cytoplasm and hydrophilic structures, such as glycogen storages and proteins. Here we used the FF of each voxel to separate the underlying lean and fat masses (Figure 5A, see “Methods” section for details). Interestingly, we observed a biphasic effect of cold exposure on supraclavicular adipose tissue mass (Figure 5B). There was an apparent decrease in the number of voxels with a high FF, most pronouncedly observed as a decrease in lipid mass on the right side of the plot (i.e., 70–100% FF). Lean mass was also decreased in this range, albeit to a lesser extent. When the left side of the plot was taken into account (i.e., voxels included in the FF range below 70%), lean and fat masses were increased to a similar extent. Both lean mass and fat mass explained a large part of the variance of the total supraclavicular adipose volume, with slight dominance of lipid mass (*R*^2^ = 0.92) over lean mass (*R*^2^ = 0.85) (Figure 5C). The discrepancy between loss and gain was quantified in the total mass variation of the tissue, where total lean mass was increased from 15.7 ± 1.6 to 17.2 ± 1.7 g (+1.5 g; *p* = 0.001) and total lipid mass in the supraclavicular depot decreased from 22.1 ± 1.9 to 21.0 ± 1.7 g (−1.2 g; *p* = 0.02) (Figure 5D).

### Tissue Energy Storages Are Decreased After Cold Exposure

**Figure 6:**
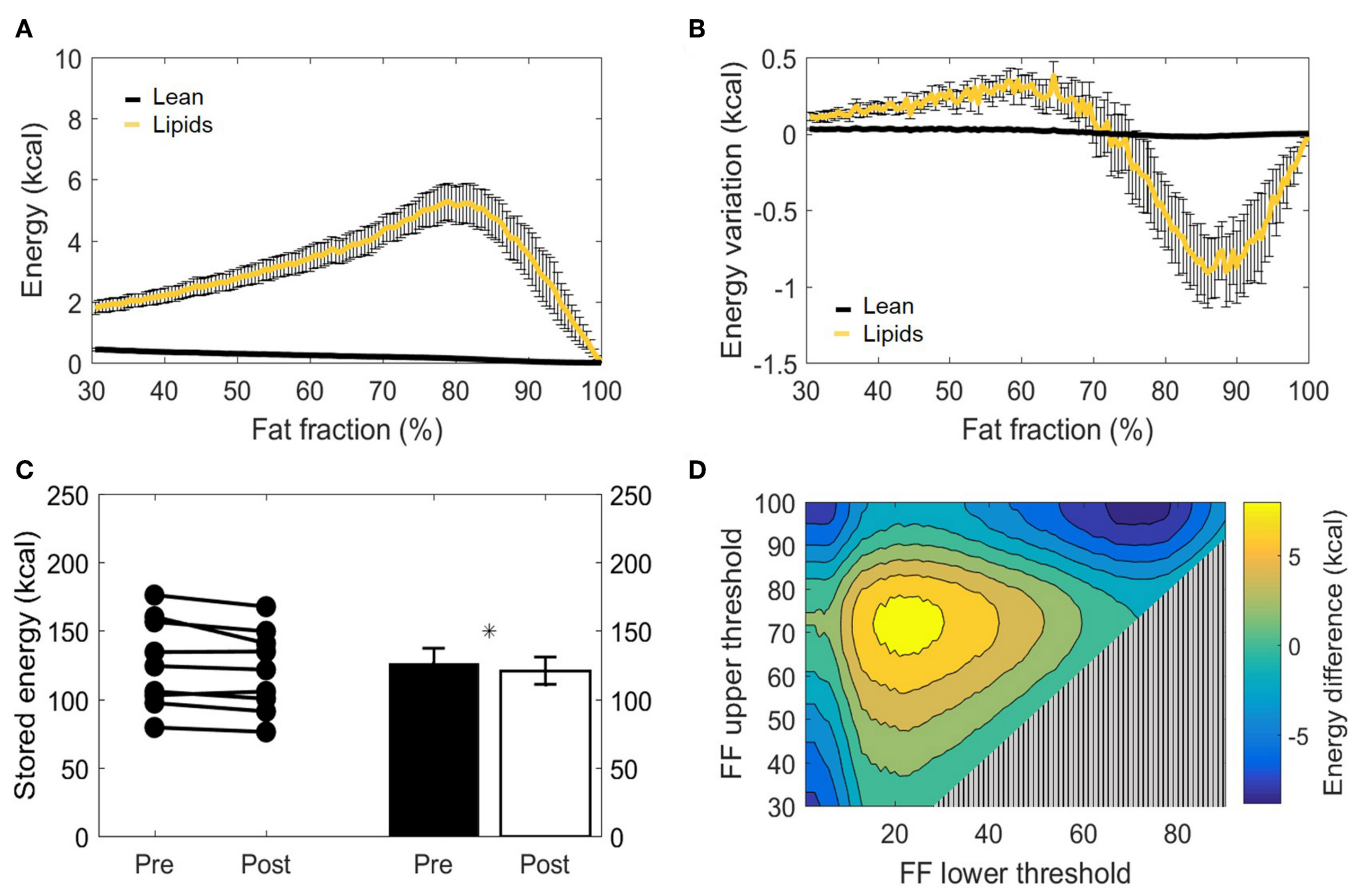
Metabolizable energy content in the supraclavicular adipose depot. Representation of energy content in the supraclavicular depot at thermoneutrality, with specific values attributed to lean tissue or lipids **(A)**. Changes in energy content attributed to lean or fat masses, represented over different fat fraction ranges **(B)**. Total energy storages (kcal) before and after cold exposure analyzed, by using the paired *t*-test **(C)**. Heatmap of the effect of different FF segmentation thresholds on estimated energy content differences after cooling. The color (second y-axis) depicts the estimated energy content difference for each lower (x-axis) and upper left (y-axis) threshold. The largest decrease in estimated energy is present with a lower threshold of 70% and no upper threshold. The triangle in the lower right corner indicates invalid FF threshold options, as we implemented a minimum FF threshold of 30% **(D)**. Data represent mean ± SEM of all participants (*n* = 9). *p < 0.05.

The main function of BAT is to convert chemical energy into thermal energy. Estimation of metabolic energy content in lean and fat masses has been validated in well-controlled experiments measuring whole-body energy intake and expenditure (38), and the concept of energy equivalence has been used to quantify the energy influx to BAT during cold exposure (40). In addition, because BAT does not contain significant amounts of bone mineral or air and the tissue water is bound to proteins, its total mass can be taken as the potential energy substrate for heat generation. Therefore, we set out to quantify the cold-induced change in energy storages. BAT is composed of a mixture of lean and lipid masses, but its chemical energy storage equivalence is largely dominated by the lipid component (Figure 6A). When analyzed from this bioenergetic perspective, the variation in lean mass previously observed by us (Figure 5B) became insignificant, as cold-induced changes in energy content attributed to lean mass was substantially lower compared to energy variations in lipid mass (Figure 6B). Here, the significant decrease in fat mass was reflected in a diminished energy storage in the supraclavicular depot, which decreased from 126 ± 11 to 121 ± 10 kcal (−5 kcal; *p* = 0.03, Figure 6C). It was noticeable that this variation was not uniform in the volume histogram, but instead there were losses in the initial high-lipid area and gains in initially leaner parts of the tissue. To better visualize this effect, a contour plot was created to represent different thresholding possibilities for the analysis of energy variation (Figure 6D). When the higher FFs of the tissue were chosen, a large decrease in energy content was inferred. On the other hand, an analysis focusing on the FF interval between 30 and 70%, for example, would have resulted in the opposite conclusion that the tissue increased its chemical energy storage after cold exposure.

### Local Assessment of the Supraclavicular Adipose Tissue FF Distribution After Cold Exposure

**Figure 7:**
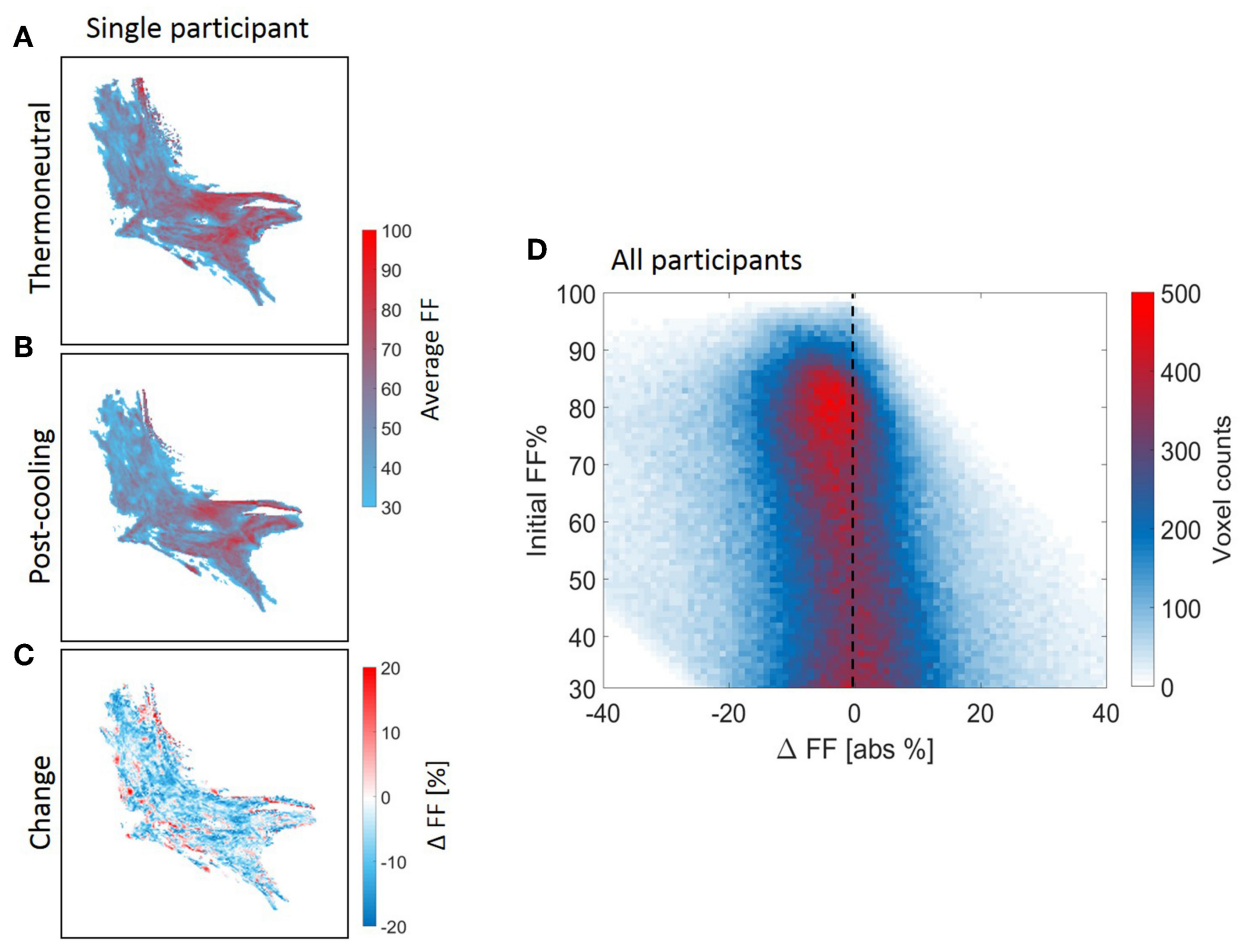
Structural heterogeneity of brown adipose tissue in the supraclavicular region during cold exposure. Example of a reconstructed fat fraction map with merged z-slices before and after cooling **(A,B)** and cold-induced change (post-minus pre) **(C)** for *n* = 1. The 2D joint voxel histogram representing variation in change in lipid content of each voxel in relation to its thermoneutral FF from the voxel-wise analysis, wherein the colors represent the number of voxels belonging to each combination **(D)** for all participants (*n* = 9). Cold colors indicate decreases in fat fraction and warm colors indicate increases in fat fraction.

Voxel-wise thermoneutral and post-cooling FF maps unveiled that the supraclavicular adipose tissue is composed of a juxtaposition of low-and high lipid zones, as exemplified in Figures 7A,B. After cold exposure, which is generally shown to decrease BAT lipid content, we found a high spatial variability in responses since several areas presented the expected reduction in lipids, while in contrast, other tissue areas increased their lipid content (Figure 7C). Lipid maps of the other eight subjects are presented in Supplemental Figure S4. Local FF changes were evaluated using a 2D joint histogram, where every voxel had its initial FF used as a reference to define the variation in FF that it underwent upon cold exposure, and the number of voxels belonging to each combination was added to represent the counts (color scale; Figure 7D). Assuming the vertical line as zero change, we observed FF changes along the entire thermoneutral FF range, with a clear increase in voxel-density in the higher FF range. To quantify this, K-means clustering was applied with the optimal cluster number equal to four. The results are shown in Supplemental Figure S5. Cluster analysis indeed revealed that for the high thermoneutral FF range, FF decreases were observed especially within cluster C1 (average thermoneutral FF: 76.0 ± 11.2%). The average FF decrease after cold-exposure that corresponded to this cluster was −3.5 ± 2.2%.

### The Association Between Supraclavicular Adipose Tissue FF and T2* on a Local Level

**Figure 8:**
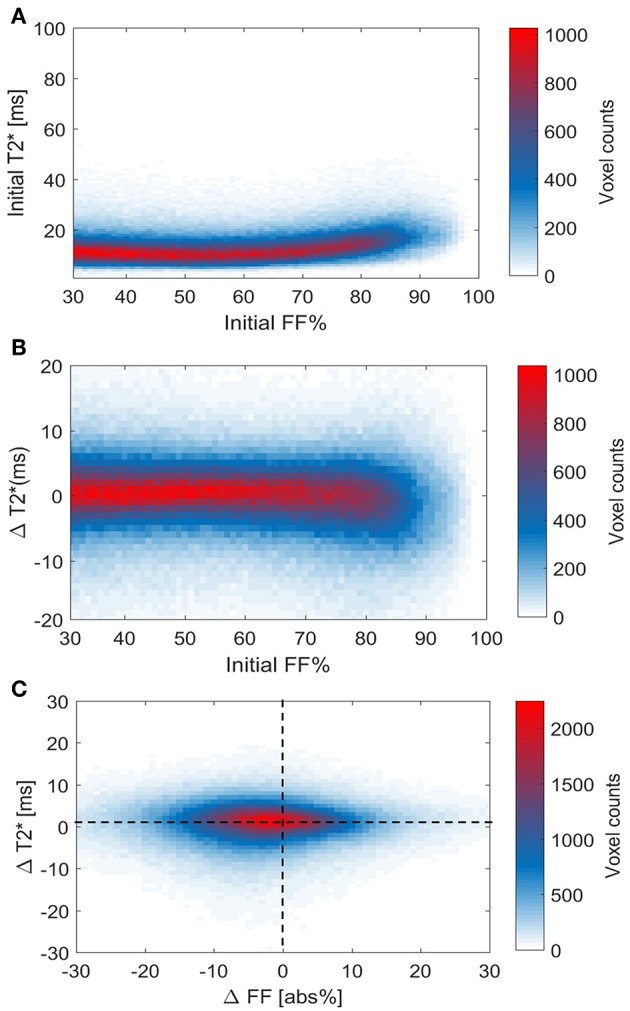
Voxel histograms representing the relation between thermoneutral values and cold-induced changes in T2* and FF. Thermoneutral measurements of T2* against thermoneutral fat fractions **(A)**. Relation between the cold-induced changes in T2* and thermoneutral fat fractions **(B)**. The association between cold-induced changes in both T2* and FF **(C)**. Data is presented as the mean of all participants (*n* = 9).

Using voxel-wise analysis, we then studied the relation of the baseline T2* relaxation time to tissue FF (Figure 8A). ${\text{T}2}_{\text{Loc}}^{*}$ values were near 10 ms at the lower FFs and circa 20–25 ms at the highest FFs. However, there was no clear relation between the baseline FF_Loc_ and ${\text{T}2}_{\text{Loc}}^{*}$ values. Also, when the cold-induced changes in ${\text{T}2}_{\text{Loc}}^{*}$ were plotted against baseline FF_Loc_, no clear association was observed (Figure 8B). Regarding the changes in T2* _Loc_ and FF_Loc_ in response to cold exposure, for most voxels FF_Loc_ decreases were accompanied by increases in ${\text{T}2}_{\text{Loc}}^{*}$ (Figure 8C). The voxel distribution was analyzed using k-means clustering. Cluster C1 included the highest voxel counts per data point (Supplemental Figure S5). For this cluster, the average ${\text{T}2}_{\text{Loc}}^{*}$ and FF_Loc_ changes were 1.4 ± 1.5 ms and −2.2 ± 4.0%, respectively.

## Discussion

In this study, we show that reductions in volume, mass and energy of the supraclavicular adipose tissue depot during cold exposure are heterogeneous and take place most prominently within lipid-rich regions of the tissue, whereas no significant changes were observed in the SAT FF. Leaner areas of the supraclavicular adipose tissue depot (defined by a low thermoneutral FF), however, tended to gain volume, mass, and energy following cold exposure. We also showed that the location and width of the FF interval can alter the apparent size and direction of cold-induced changes of MRI-derived parameters used for BAT analysis. The maximum FF change to the entire supraclavicular adipose depot was obtained by implementing a 34–100% FF range. Finally, local changes in FF occurred over the entire thermoneutral FF range (30–100%) in both directions (i.e., increase and decrease).

### The Upper FF Threshold Range for BAT FF Analysis

The classical distinction between unilocular WAT and multilocular BAT suggests that a clear division based on FF should exist between both tissues. From this perspective, the range where FF is higher than 70% has previously been assumed to be above the BAT threshold (52). For this reason, we found it remarkable that, in our results, these high-lipid areas of the supraclavicular adipose depot actually showed the largest decrease in lipid and energy content after cold exposure, which is in agreement with recent findings (25). These data suggest that, in fact, one should not use 70% as an upper threshold, and voxels showing up to 100% FF should be used in the analysis [e.g., as performed in (22, 53)]. Unfortunately, it was not possible to infer whether these regions comprised unilocular white adipocytes that partially donated their lipids for combustion by surrounding “leaner” brown adipocytes. Alternatively, this region could englobe unilocular UCP1-expressing cells capable of thermogenesis. In both scenarios, the lobular distribution of high-fat zones, intercalated by regions of lower lipid content, suggests that human BAT should be taken as a morphologically diverse organ, and care should be used before excluding areas from its analysis. T2* analysis did not provide any additional information in establishing lower and upper FF thresholds for BAT segmentation.

### The Lower FF Threshold Range for BAT Analyses of FF and Volume

Both the global and local analyses showed that changes occurred across the entire baseline FF range (30–100%), with the greatest apparent FF decrease when using a 34–100% FF range. The largest FF decrease we observed (i.e., 3.5%) is in the range of values reported in literature (19, 21, 23), but also much smaller (22) and larger decreases (25) have been reported. This could be due to the use of different thresholds, but also differences in the cooling protocols can play an important role (54, 55). Raising the lower threshold above 34% decreases the extent of FF differences upon cold exposure, as we excluded voxels that fell below the threshold in both the thermoneutral and post-cooling scan in order to avoid partial volume artifacts and to enable volumetric analysis. For example, when a lower FF threshold of 70% is used, voxels below 70% FF are excluded in both the thermoneutral and post-cooling ROIs. Hence, regions in the post-cooling ROIs that shifted from high thermoneutral FFs (>70%) to FFs below 70% upon cold exposure are excluded, but are still present in the thermoneutral ROIs. These lower FF regions can, therefore, not contribute to the reduction of FF_Glob_ in the post-cooling ROIs. A recent report where the use of FF thresholds were also explored showed an opposite effect, as a larger effect on FF was shown using a 50% threshold compared to a 40% threshold. In their approach, FF thresholds were only applied to the thermoneutral ROIs (25), which could have enabled measuring larger FF differences with increasing lower thresholds because voxels in the post-cooling scans were not excluded. This indicates that care should be taken before excluding low-lipid areas from the analysis.

The total estimated BAT volume showed an opposite trend compared to FF, where increasing the lower FF threshold enlarged the differences. This is expected, as most prominent volume reductions take place above a FF of about 70%.

### On the Heterogeneity of Human Supraclavicular Adipose Tissue

In this work, we expanded the idea of supraclavicular adipose tissue heterogeneity by visualizing its structure, its complex distribution of lipids and described the variations in the lipid content (increased and decreased in the same depot) after cold exposure. These data strongly suggest that BAT acutely modulates lipid influx and combustion divergently, here exemplified by the supraclavicular areas that gained lipids after thermogenic activation by cold exposure, which was also shown in a recent study (25). This example goes against expectations of BAT only decreasing its lipid content, an idea so broadly accepted that the loss of lipids during cooling has been used as a condition sine qua non for the identification of BAT (23). The guiding factors behind the cold-induced lipid gain in some BAT areas are unclear. We speculate that an increase in lipids is also possible due to *de novo* lipogenesis taking place after glucose uptake (56).

### Mass Quantification Within the Supraclavicular Adipose Depot

In the present work, we estimated the absolute amounts of lean and fat masses within the supraclavicular adipose depot. This provided the insight that, at least in our lean young subjects, fat and lean masses (conceptualized as representing the lipid storages and the metabolically-active components of the tissue, respectively) had a high linear correlation with total tissue volume. Therefore, we assume that estimated BAT size in its simplest measure is likely to be correlated to its total potential thermogenic function. The cold-induced decrease in total lipid mass seen in our study was expected because of the thermogenic activation of BAT, which leads to increased β-oxidation (57, 58), and is in agreement with other imaging studies using FF as an outcome (19, 21, 25, 59). This was accompanied by an increase in lean mass, which is unlikely to be caused by acute protein synthesis, since our entire experiment took place in a few hours. The increase in blood perfusion expected to happen in BAT during cold exposure (2, 22, 60–63) could contribute to an increase in water signal. However, it was recently postulated that FF reductions immediately after cold-exposure are too large to be solely achieved by increasing the blood volume fraction (25). Additionally, cold-induced FF decreases were shown to be maintained even after reheating the subject, which does not coincide with the fast dynamics of perfusion (19, 25). These findings support the rationale that the observed decrease in lipid mass and increase in lean mass are prominently caused by the intracellular lipid depletion in brown adipocytes. This results from the very general classification of lean mass as a collection of structures richly bound to water, which makes it susceptible to acute changes in hydration levels (64).

In a broader context of metabolic studies, lean mass is generally understood to be the major determinant of whole-body basal metabolic rate. Because the contribution of specific organs to the whole-body basal metabolic rate can be estimated based on their total mass (65–67), we predict that the evaluation of the specific lean mass of organs (such as performed in our study) may contribute to the generation of better allometric models to infer on organ-specific metabolic rates and their influence on whole-body energy expenditure.

### Energy Variation Following Thermogenic Activation

The supraclavicular adipose tissue composition analysis demonstrated the dominance of fat mass on energy dynamics during cold exposure. Critically, although lean mass comprised almost half of the tissue, even significant variations in its mass are not likely to play a major role in metabolic energy storage. We can only speculate on whether this reflects a decreased volume of larger lipid droplets due to combustion, increased lipid droplet formation due to lipid uptake from the bloodstream, or a combination of both phenomena. Based on the principle of energy conservation, it can be postulated that, if the nutrient uptake by the tissue perfectly matches its combustion rates, the fat energy loss and gain within different FF of the organ will be equal to zero. Results differing from zero can be interpreted as an uncompensated or overcompensated lipid (or glucose) uptake from the bloodstream (in relation to BAT expenditure during cooling). Most importantly, while our setup did not allow us to estimate the total energy flux of the tissue, it did provide an important conceptual milestone for the quantification of BAT-specific energy expenditure. Because expenditure can be estimated based on combinatory measurements of glucose and lipid uptake and variations in tissue composition, we predict that the method employed in our study [allied to energy uptake estimations by Virtanen et al. (40)] will make it possible to finally infer concerning the energy combusted by BAT during activation and to more accurately quantify the specific contribution of BAT depots to whole-body metabolism.

### General Conceptual Applications of the Method

The application of the bioenergetic framework presented here is not confined to the analysis of BAT during cooling. It can also be used for the analysis of metabolic content in any tissue where energy storages are crucial for pathophysiological processes. These include muscles, where changes in energy availability can modify the long-term maintenance of the mass, as well as the liver, where excessive energy storages in the form of lipid droplets are thought to be causal to insulin resistance and metabolic diseases.

### Limitations

We could only partially infer about the dynamic changes in tissue composition due to the limited number of time-points, i.e., one before and after cooling. Dynamic scans would possibly provide more insights into changes in lipid composition within the supraclavicular adipose depot. In our study, we used six echoes for the mono-exponential T2* fit. Recently, a study has shown that the accuracy of the fit enhances with increasing echo number (26), and therefore in future studies the echo number will be increased to improve T2* measurement in BAT. We did not perform respiratory triggering in acquisition, which could have led to motion artifacts. We mitigated this by using a 3 × 3 smoothing kernel after registration. In addition, a recent study that employed similar MR methodology without respiratory triggering demonstrated an error of less than one pixel after image registration (25). This study included a relatively homogeneous study population (young, male, healthy, lean white Dutch natives). Therefore, caution should be used when extrapolating our results to a more general population. Instead, it is recommended to assume our results as representing those of a control population and as a demonstration of methodological possibilities to track alterations in obesity, disease or drug testing. The extent of cold-induced FF changes that have been reported in literature and in this study are quite modest. It has been also shown that there is only a small, albeit statistically difference in supraclavicular FF between individuals with and without BAT activity on [^18^F]FDG PET-CT (68). BAT activity assessed by glucose uptake in PET/CT and by FF differences upon cold exposure, however, are not measuring the same exact response. This is not unexpected, as in [18F]FDG PET glucose is used as a tracer, while in fat-water MRI we are assessing the fat content directly. Future studies including multiple MR sequences each tuned to a different aspect of physiology will hopefully further elucidate this issue.

## Conclusion

The supraclavicular adipose depot in humans is highly heterogeneous with respect to basal lipid content, and lipid-rich areas are intercalated with lipid-poor regions. After thermogenic activation by cooling, areas of the tissue with a high FF tend to lose more lipids, while an increase in mass is noticeable in the leaner regions. Cold-induced loss of metabolic energy is more noticeable in the high 70–100% FF range. Overall, cold exposure decreases absolute lipid mass and tissue energy content, which is associated with an increase in lean mass, but does not significantly change tissue volume. Due to variability of the supraclavicular adipose depot when responding to cold exposure, the choice of MRI thresholding highly affects the estimated magnitude and direction of changes. Overall, we found that by increasing the lower FF threshold level, global FF differences became less pronounced, whereas estimated BAT volume differences became larger in magnitude. This emphasizes that the selection of FF threshold levels can affect parameters differently.

## Data Availability Statement

Data is available from the corresponding author on reasonable request.

## Ethics Statement

The studies involving human participants were reviewed and approved by METC Leiden-Den Haag-Delft, The Netherlands. The patients/participants provided their written informed consent to participate in this study.

## Author Contributions

KN and LJ designed the study, collected and analyzed the data, and revised the manuscript for intellectual content. GA-V conceptualized, analyzed and interpreted the data, and drafted the manuscript. AS reanalyzed the data and drafted the manuscript. JB analyzed and interpreted the data, developed the MRI acquisition protocol and post-processing algorithms, and drafted and revised the manuscript for intellectual content. OD contributed to the methods (data analysis) and revised the manuscript for intellectual content. JE contributed to the post-processing algorithms. TR analyzed and interpreted the data. AW revised the manuscript for intellectual content. HK, PR, and MB conceptualized and designed the study, interpreted the data, contributed to the discussion, reviewed and edited the manuscript.

### Conflict of Interest

The authors declare that the research was conducted in the absence of any commercial or financial relationships that could be construed as a potential conflict of interest.

## Abbreviations

BAT: Brown adipose tissue
FF: Fat fraction
FF_Glob_: Supraclavicular adipose tissue fat fraction estimated by global analysis
FF_Loc_: Fat fraction on a voxel-level
FF_SAT_: Fat fraction of the subcutaneous adipose tissue depot
MRI: Magnetic resonance imaging
ROIs: Regions of interest
T2*: Mono-exponential effective transverse relaxation time
${\text{T}2}_{\text{Glob}}^{*}$: Mono-exponential effective transverse relaxation time estimated by global analysis
${\text{T}2}_{\text{Loc}}^{*}$: Mono-exponential effective transverse relaxation time estimated on a voxel-level
Vol_BAT_: Estimated BAT volume
WAT: White adipose tissue
SAT: Subcutaneous adipose tissue.
