# Supplementary material for: Human Brown Adipose Tissue Estimated With Magnetic Resonance Imaging Undergoes Changes in Composition After Cold Exposure: An in vivo MRI Study in Healthy Volunteers
Source: Front Endocrinol (Lausanne). 2020 Jan 9;10:898. doi: 10.3389/fendo.2019.00898 (PMC6964318; doi:10.3389/fendo.2019.00898)
Supplement: Supplementary file 1 [file Data_Sheet_1.pdf]

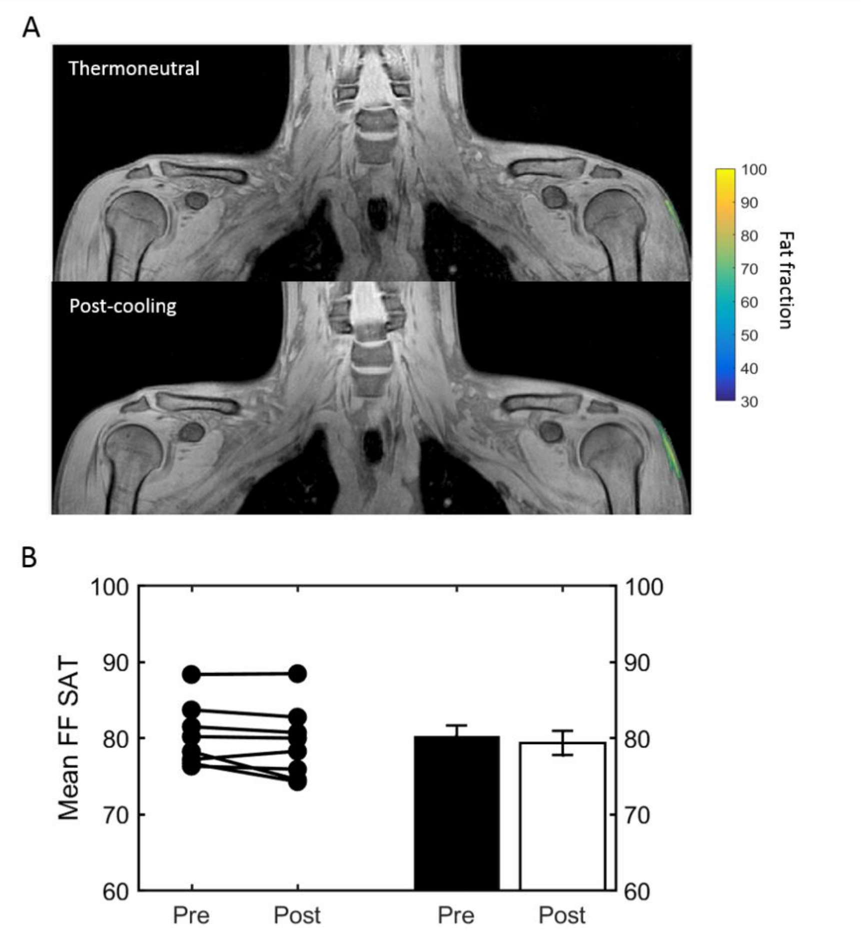


**Supplemental Figure S1. Deltoid SAT ROI delineation and SAT FF (FFSAT) analysis**
The SAT ROI in the thermoneutral scan (top) and post-cooling scan (bottom) (**A**). Lipid content in the SAT region is color-mapped over a 30–100% FF range. SAT FF before and after cooling (**B**). Data are represented as mean±SEM for n=8. Data analysis was performed using the paired t-test.

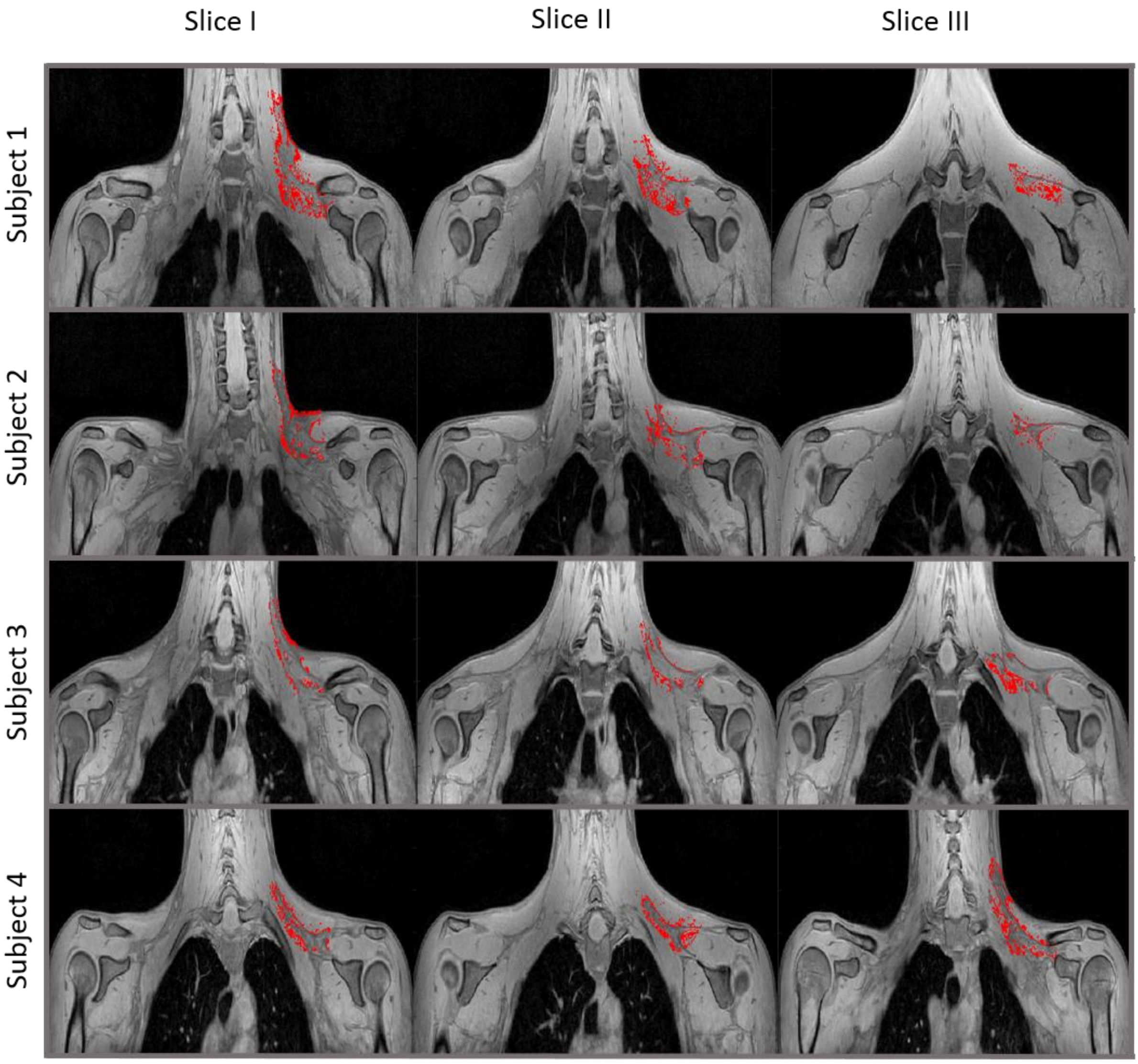


**Supplemental Figure S2. Localization of low FF voxels (10–30%) in the supraclavicular adipose depot**

Voxels within the 10–30% FF range are shown for a selection of the group (n=4). For each participant three slices, ordered in the anterior-posterior direction, are presented. Only voxels above 10% are shown to emphasize the boundaries of the supraclavicular depot.

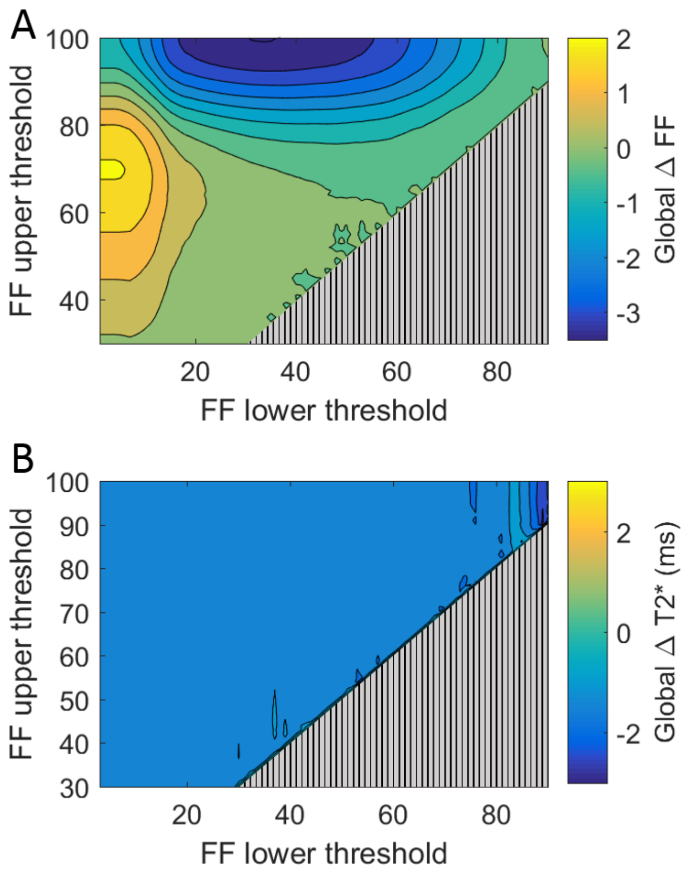


**Supplemental Figure S3. Effects of different FF thresholding options on cold-induced global FF and T2* changes**

Heatmap of the effect of different FF segmentation thresholds on the FF and T2* changes after cooling. (A)The color (second y-axis) depicts the FF for each lower (x-axis) and upper left (y-axis) FF threshold. The largest FF decrease is present with a lower threshold of 34% and no upper threshold. (B) The color (second y-axis) depicts the T2* for each lower (x-axis) and upper left (y-axis) threshold. The largest T2* decrease is present with a lower threshold of 90% and no upper threshold, however this was not found to be significant. The triangle in the lower right corner indicates invalid FF threshold options, as we implemented a minimum FF threshold of 30%. Data represent the mean of n=9 subjects.

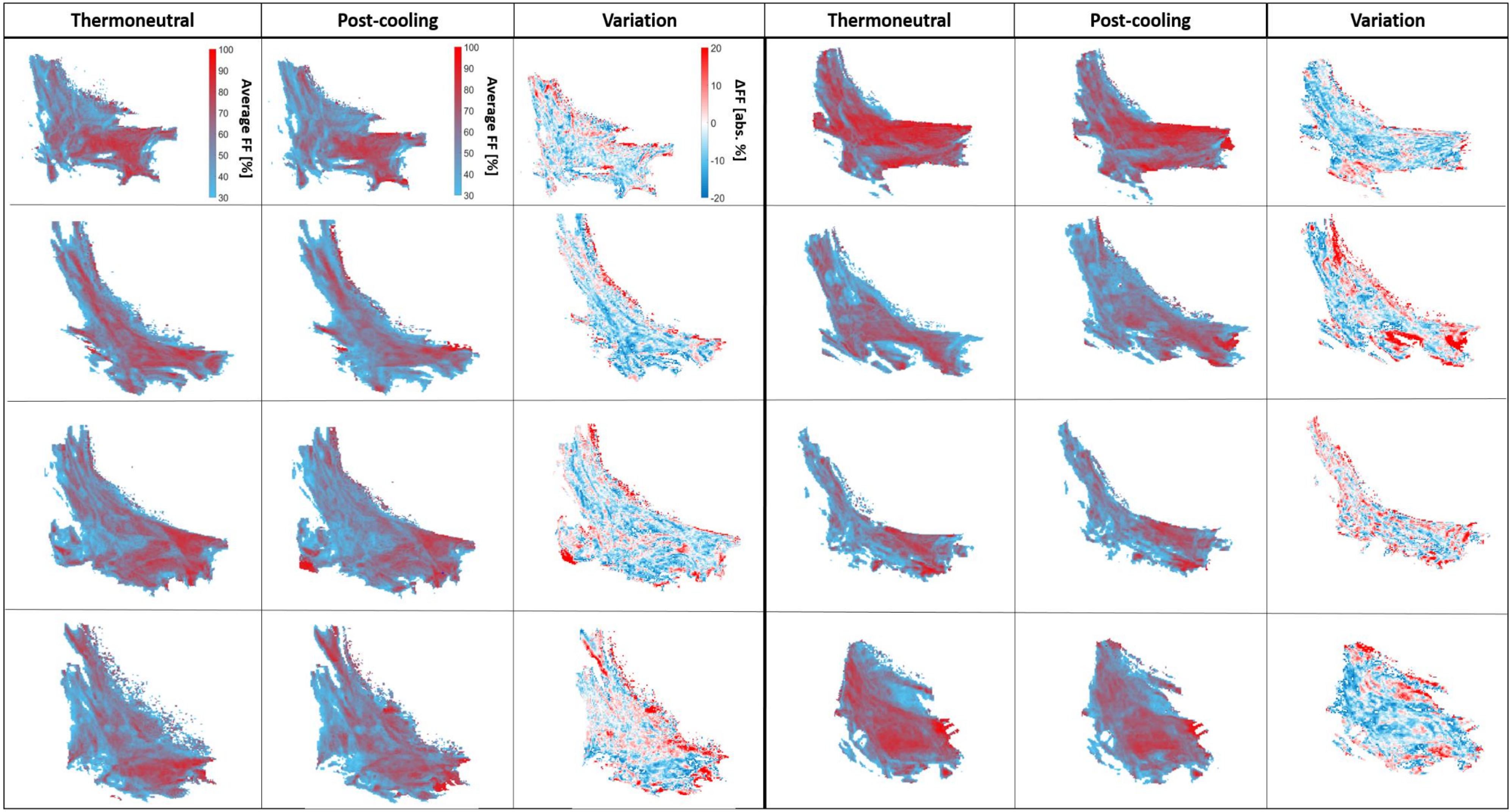


**Supplemental Figure 4. Fat fraction maps of the supraclavicular adipose depot with merged z-slices**
Average FF value of voxels containing the same ($x$,$y$) position was extracted to construct the FF and FF variation maps. Voxels below 30% FF were thresholded in order to avoid inclusion of non-fatty tissue and to minimize partial volume interference. For thermoneutral and post-cooling FF maps, the red color indicates high lipid regions and the blue color — low-lipid regions. Cold-induced changes are represented by the red color for increased lipid content and the blue color for decreased lipid content.

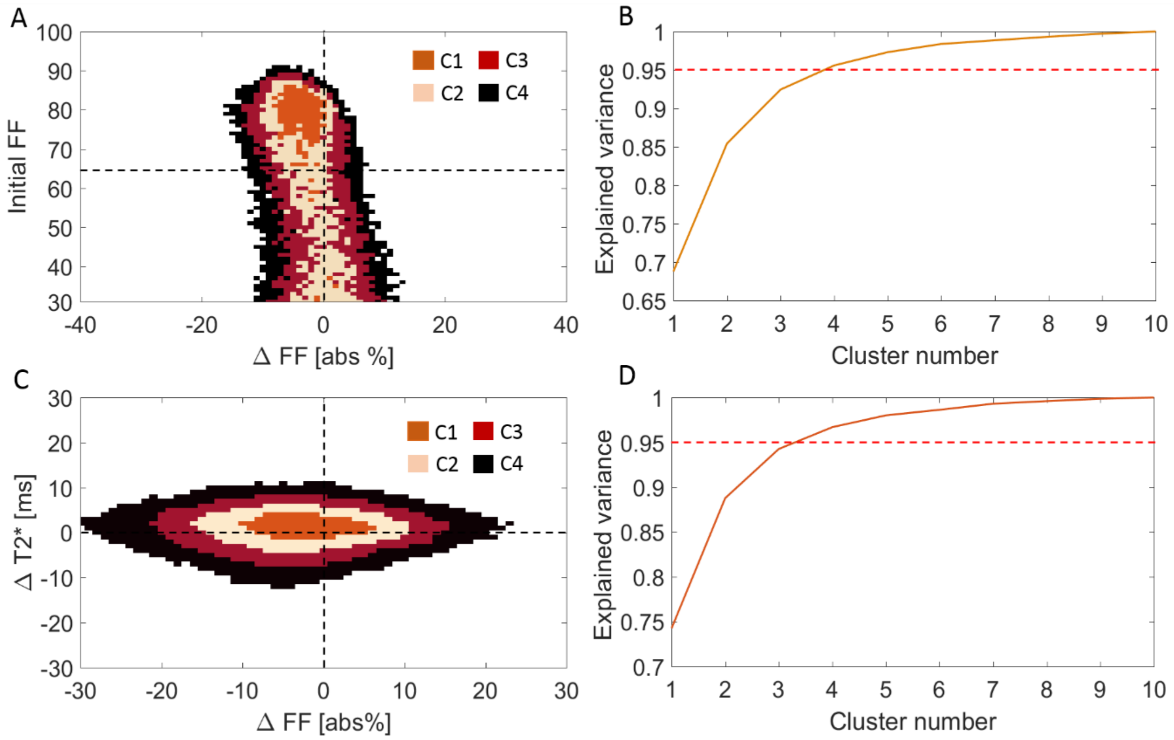


**Supplemental Figure S5. K-means cluster analysis for local assessment of supraclaivuclar adipose tissue baseline $FF_{Loc}$ and cold-induced $FF_{Loc}$ and $T2^*_{Loc}$ changes**

The joint voxel distribution from Fig. 7D partitioned into four clusters (**A**). The optimal number of clusters was obtained by visual inspection of the explained variance plot (cut-off 95%): it was noted that the rise in explained variance became increasingly less after the fourth cluster (**B**). Clustered joint voxel distribution from Fig. 8C (**C**). Also for this analysis four clusters were used, basedon the explained variance plot shown in (**D**). Data represent the mean of n = 9 subjects.
